# A review of emerging technologies, nutritional practices, and management strategies to improve intramuscular fat composition in beef cattle

**DOI:** 10.1080/10495398.2024.2388704

**Published:** 2024-08-12

**Authors:** Belete Kuraz Abebe, Jianfang Wang, Juntao Guo, Hongbao Wang, Anning Li, Linsen Zan

**Affiliations:** a College of Animal Science and Technology, Northwest A&F University, Yangling, Shaanxi, People’s Republic of China; b Department of Animal Science, Werabe University, Werabe, Ethiopia; c National Beef Cattle Improvement Center, Northwest A&F University, Yangling, Shaanxi, People’s Republic of China

**Keywords:** Cattle, emerging technologies, intramuscular fat, nutrition, management

## Abstract

The flavour, tenderness and juiciness of the beef are all impacted by the composition of the intramuscular fat (IMF), which is a key determinant of beef quality. Thus, enhancing the IMF composition of beef cattle has become a major area of research. Consequently, the aim of this paper was to provide insight and synthesis into the emerging technologies, nutritional practices and management strategies to improve IMF composition in beef cattle. This review paper examined the current knowledge of management techniques and nutritional approaches relevant to cattle farming in the beef industry. It includes a thorough investigation of animal handling, weaning age, castration, breed selection, sex determination, environmental factors, grazing methods, slaughter weight and age. Additionally, it rigorously explored dietary energy levels and optimization of fatty acid profiles, as well as the use of feed additives and hormone implant techniques with their associated regulations. The paper also delved into emerging technologies that are shaping future beef production, such as genomic selection methods, genome editing techniques, epigenomic analyses, microbiome manipulation strategies, transcriptomic profiling approaches and metabolomics analyses. In conclusion, a holistic approach combining genomic, nutritional and management strategies is imperative for achieving targeted IMF content and ensuring high-quality beef production.

## Introduction

In the beef industry, intramuscular fat (IMF) composition in beef cattle is a significant factor.^1^^,^^2^ It impacts the flavour characteristics of beef and plays a key role in its palatability.^3^^,^^4^ Higher levels of intramuscular fat percentage (IMF%) intensify sweet and grilled or roasted flavours while reducing off-flavours, leading to enhanced overall flavour intensity and evaluation scores. However, excessively high levels of IMF% can diminish flavour intensity and lead to less appealing sensory qualities.^4^ Research has indicated that vitamin A can reduce IMF in cattle steers.^5^ The process of IMF deposition involves changes in the microRNA transcriptome and gene expression, including preadipocyte differentiation followed by increased expression of lipogenic genes.^6^ The quantity of IMF, also known as marbling, influences the grading and pricing of carcasses’ quality grades. Key adipogenic marker genes play a crucial role in transcriptional regulation for IMF deposition, specifically in Qinchuan beef cattle.^7^ Understanding and enhancing IMF composition is essential for improving meat production standards and achieving high-quality traits within the beef industry.

The economic importance of high-quality beef products with optimal marbling is rooted in consumer demand. Consumers are willing to pay a premium for beef that has excellent quality grades, such as domestically labelled premium-quality beef.^8^ This suggests a potential market for higher-quality beef grade, such as Ecuador.^9^ However, perceptions of meat quality and preferences can vary between countries. Consumers in Turkey may not view higher degrees of marbling as an indicator of superior meat quality.^9^Consumers in developing countries may prefer their meat to be cooked to higher levels of doneness. Hence, it is crucial to comprehend consumer preferences and perceptions in order to enhance satisfaction and cater to market demands in these regions.^10^

IMF is a critical element in determining the nutritional value of beef such as flavor, juiciness, and tenderness; on the other hand, a low-fat content results in less tasty meat.^11–16^ The development of IMF in cattle begins after birth and continues throughout the animal’s life. Genomics, dietary and handling factors all have a significant impact on IMF accumulation.^17–19^ The content of IMF in beef, including skeletal muscle tissues is among the most prominent factors affecting the lusciousness and beef quality.^17^^,^^20^^,^^21^ IMF is critical for improving beef flavour and palatability and is more abundant in the late phases of animal development.^4^^,^^22^^,^^23^ The presence of IMF in beef is among the crucial issues associated with consumer perception of beef eating quality.^16^^,^^23^^,^^24^ The interaction of muscle with IMF composition has an influence on consumer rankings of beef flavor.^23^^,^^25–27^

Consumer preferences for premium beef products are leading to a focus on improving IMF composition. This is driven by the desire for beef with a higher IMF content, which is linked to better tenderness, juiciness, and flavour. It’s also considered healthier due to higher levels of unsaturated fatty acids. However, there is a limited understanding of IMF deposition beyond the carcass stage. Research on genetic factors influencing IMF content and developing prediction models for its detection is underway.^28^ Additionally, educating consumers about the nutritional value of beef and its importance in purchasing decisions can lead to a willingness to pay more for steaks with improved fatty acid composition.

Genotype and production system have a strong influence on the composition of IMF.^29^^,^^30^ Genetic factors influence the composition of beef fatty acids, while the fatness of specified breed affects the ratio of polyunsaturated fatty acid to saturated fatty acid.^31–33^ The composition of lipid and fatty acid profile of IMF in beef might be operated on the basis genetics, feeding time, and finishing diet.^34–36^ The expression of marbling is caused by maintained fat synthesis and IMF accumulation begins shortly after birth and continues throughout the animal’s growth.^37–39^ Traditional animal breeding procedures rely on recording animals based on their based on phenotypic measurements to determine their breeding value. However, IMF content and muscle mass can only be directly measured after slaughter. Thus, these procedures may not yield accurate IMF content and muscle mass breeding values for live animals.^40^^,^^41^

Beef is a substantial source of proteins, minerals, and B-complex vitamins, but it also has a huge amount of saturated fatty acids.^30^ High intake of saturated fatty acids augments the level of serum cholesterol and low-density lipoproteins which increases the risk of cardiovascular.^42^ Moreover, bovine fat mostly contains saturated fatty acids like myristic (C14:0), palmitic (C16:0) and stearic (C18:0).^43^ Notably, C14:0 has the capacity to boost serum cholesterol levels 4 to 6 times more than C16:0.^44^

For now and the future, the new effort on finding genes and other factors responsible for the IMF accumulation in beef is crucial for enhancing healthy fatty acids while lowering saturated fatty acids in visceral and subcutaneous fat.^30^^,^^45^ In addition, the amount of fatty acids in beef is crucial because it affects the palatability of the beef and human well-being. Nevertheless, the effects of harmful fatty acids on human health have received less attention in various regions of the world.^32^ However, a number of studies have been done in various parts of the world to determine a single factor that influences the level of IMF accumulation in beef cattle. Conversely, the combined influence of emerging technologies as well as nutritional and management factors that could improve the accumulation of IMF in beef cattle has not been thoroughly studied. Therefore, the aim of this paper was to provide insight into emerging technologies, nutritional and management strategies to improve intramuscular fat composition in beef cattle. The specific objectives were: (1) To identify and summarize the current research on emerging technologies for improving IMF composition in beef cattle; (2) To assess the effectiveness of various nutritional strategies for improving IMF composition in beef cattle and (3) To examine the impact of different management strategies on IMF composition in beef cattle. The review gathered data from a range of secondary sources, such as relevant books, academic articles, and online materials. Non-English-language studies and those with insufficient data were excluded. Information was then presented in line with the objectives.

## Management practices

Management techniques, including multi-trait selection indices for feeder cattle and fertile replacement heifers, lowering the age at slaughter, and raising carcass weight, can be used to increase the sustainability of beef production.^46^ The accumulation of IMF in beef cattle can be significantly impacted by innovative management techniques. These procedures may involve adjustments to animal care procedures, food schedules and environmental factors.^47^ For instance, studies have demonstrated that allowing cattle to graze for extended periods of time or enabling them access to pasture might enhance the accumulation of IMF in beef cattle.^18^^,^^48^ Utilizing low-stress handling approaches is another management strategy that may have an impact on IMF accumulation. Cattle managed with low-stress approaches have higher levels of IMF than cattle treated with traditional methods.^18^This might be because low-stress handling results in less stress hormone production, and stress hormones might have a negative effect on cattle’s ability to store fat. Environmental factors can affect the deposition of IMF in beef cattle, in addition to feeding and handling procedures. Research has demonstrated, for instance, that beef cattle can accumulate more IMF when exposed to low temperatures.^18^ This might be because animals demand more energy when it’s cold outside, which can encourage the deposition of IMF. Overall, these management techniques may significantly affect the deposition of IMF in beef cattle.^18^

Successful management practices such as the castration of bulls and tailored feeding schedules for different growth stages, have been demonstrated to enhance IMF accumulation in beef cattle.^17^^,^^49–52^ Conversely, inadequate handling, transportation, and pre-slaughter management can negatively affect meat quality. Stressful handling and poorly planned abattoirs can elevate the pH of meat and shorten its shelf life.^17^^,^^49^^,^^50^^,^^53^ Thus, proper breeding, feeding and management techniques that improve meat quality are vital for enhancing the viability and competitiveness of beef cattle agribusiness.

### Animal handling techniques

The manner in which animals are handled plays a significant role in how much IMF is deposited in beef cattle. Stressful handling techniques can raise cortisol levels, which can impair adipose tissue metabolism and inhibit IMF deposition.^38^ According to research, beef cattle with mild management techniques had higher IMF levels. For instance, cattle treated quietly and kindly have higher IMF levels than cattle handled loudly or forcefully.^54^ Similar to humans, cattle treated in a low-stress way during transportation had higher levels of IMF than cattle handled in stressful circumstances.^55^ Proper animal care can lessen stress in addition to enhancing feed intake and nutrient absorption, which might result in an increase in IMF deposition. For instance, calves handled delicately during feeding consumed more feed and gained more weight than cattle handled forcefully.^56^ In general, animal handling is a significant component that may have an effect on the IMF deposition in beef cattle. Stressful handling techniques can result in lower IMF levels, whereas gentle handling techniques can boost IMF levels.

### Weaning age

In cattle production, weaning age is a critical factor in determining the accumulation of IMF.^57^^,^^58^ Early weaning, which involves removing calves from their dams before they reach seven months of age, has been found to be a potential technique for promoting beef IMF composition.^59^ Studies have shown that premature weaning in combination with large amounts of energy feeds can lead to higher feed efficiency, average daily gain, hot carcass weight, and percentage of fat in breeds such as Angus.^60^ For instance, calves weaned early at 3.5 months old and fed a large amount of concentrate feeds for nearly 5 months had higher HCW and IMF than calves that were weaned normally at 8.4 months old and left to graze on pasture with their dams.^61^ This is because early weaning in conjunction with a high-energy diet activates the PPAR and C/EBP genes, leading to precocious preadipocyte differentiation and fat storage.^62^

Weaning age has an impact on IMF deposition in beef cattle. Younger calves tend to have a greater intensity of response to weaning stress, which can affect IMF deposition. Calves weaned at 30 days of age showed a higher percentage of extreme vocalization compared to those weaned at 75 or 180 days of age.^17^ Additionally, the age at which cattle are weaned can influence IMF deposition in different muscles. A study found that there was a difference in IMF deposition in the longissimus dorsi muscle throughout the study period, but no significant differences were observed between day 0 and day 56 or between day 84 and day 140.^63^ Manipulating IMF deposition through developmental programming during the foetal and neonatal periods can also impact IMF deposition in later life.^64^ Overall, weaning age can affect IMF deposition in beef cattle, and further research is needed to fully understand the relationship between weaning age and IMF deposition.

### Castration

Castration of male calves has been found to enhance the accumulation of IMF in various breeds of cattle.^65^^,^^66^ This is due to the fact that castration increases lipid absorption and lipogenesis while decreasing lipolysis, which leads to an increase in the IMF content.^67^ Studies have shown that castration during weaning can lead to higher IMF content in breeds such as Nellore, while late castration can have the opposite effect.^68^ The timing of castration can therefore have an impact on the level of IMF accumulation in beef cattle.^69^^,^^70^ In addition to enhancing the quality of beef through greater fat accumulation, castration also has other benefits. It can reduce aggressive and sexual behaviour in bulls, which makes handling and management easier.^71^^,^^72^ Castration has also been found to up-regulate PPAR and FABP4 gene expression, which further enhances the quality of beef.^71^^,^^72^

Castration of bulls has been found to increase IMF deposition in beef cattle. Transcriptomic analysis of the longissimus thoracis muscle (LT) in bulls and steers revealed differential gene expression associated with IMF accumulation following castration.^73^ Weighted gene co-expression network analysis (WGCNA) identified modules and hub genes involved in fat deposition, including *TCAP*, *MYH7*, and *TNNC1.*^74^ Castration was also found to upregulate the bone morphogenetic protein (BMP) signalling pathway, leading to increased adipogenic gene expression and IMF deposition.^75^ Additionally, management factors such as castration and nutritional factors such as fat metabolism and vitamin levels were found to affect IMF deposition.^17^ Ultrasound measurements of carcass traits in Akaushi cross steers showed differences in IMF deposition in various muscles, with a positive increase observed throughout the study.^63^ Overall, these findings highlight the impact of castration on IMF deposition in beef cattle and provide insights into the molecular mechanisms and factors influencing this process.

### Breed and sex

One of the most significant elements affecting the IMF composition of beef cattle is breed. Breeds like Angus and Wagyu are renowned for having higher marbling levels than Holsteins.^76^^,^^77^ Changes in the adipose tissue metabolic processes and the hormonal controls of fat deposition led to an increase in IMF. Gender also influences the accumulation of IMF, with females typically containing higher levels of IMF than males.^78^^,^^79^ In general, producers and consumers alike should be aware of the effects of breed, age, and sex on IMF composition because they can affect the nutritional worth of beef products.

Heifers typically have higher amounts of fat than castrated males, and both typically have higher levels of fat than intact males when comparing beef animals with similar carcass weights.^80^ However, when comparing animals with comparable levels of fatness, gender differences become less important.^80^ Furthermore, muscles from the mature cows have higher levels of fat than muscles from bulls bred in confinement or on grassland.^81^

Breed selection and sex have a significant impact on IMF deposition in beef cattle. Japanese Black cattle has the highest IMF content, followed by Korean cattle (Hanwoo).^74^ Castration of bulls increases IMF deposition in most cattle breeds.^39^ Management factors including weaning age, castration, slaughter weight and age, and environmental conditions also affect IMF deposition.^82^ Nutritional factors such as fat metabolism, feed digestion and absorption, and vitamin levels play a role in IMF deposition.^83^ The tissue network in lipid metabolism, particularly in the longissimus dorsi muscle (LM), is crucial for IMF deposition. Manipulating IMF deposition through developmental programming and applying ‘omics’ fields like epigenomics, metagenomics and metabolomics can provide insights into the molecular mechanisms of IMF deposition. Overall, a combination of genetic and management strategies, along with proper nutrition, can be used to manipulate IMF deposition in beef cattle.

### Environmental variables

The accumulation of IMF in beef cattle can be considerably influenced by environmental conditions. The metabolism and synthesis of fatty acids are influenced by variables like temperature, humidity, and feed.^84^^,^^85^ This has an impact on the quantity and quality of IMF in beef. One of the environmental elements that have the greatest impact on IMF deposition in beef cattle is temperature. High ambient temperatures have been found to reduce the amount of IMF in beef by decreasing the activity of lipogenic enzymes and increasing fatty acid oxidation.^86^^,^^87^ Another environmental element that can affect IMF deposition in beef cattle is humidity. Low IMF content in beef can result from heat stress in cattle brought on by high humidity.^86^ In summary, environmental variables like temperature and humidity can have a big impact on IMF deposition in beef cattle. Producers may optimize beef quality and production by having a thorough understanding of these elements.^86^

Environmental variables have a significant impact on IMF deposition in beef cattle. Factors such as nutrition, management practices, and weather events can influence IMF deposition.^17^^,^^74^ Nutritional factors, including fat metabolism, feed digestion and absorption, and vitamin levels, play a crucial role in IMF deposition.^63^ Additionally, environmental conditions, such as temperature and weather events, can affect IMF deposition in cattle.^88^ Furthermore, the study of water stress index (WSI) and carbon footprint (CF) in the beef cattle fattening industry (BCFS) reveals the environmental and economic impact of the industry, indicating the need for mitigation strategies to ensure sustainability.^89^

### Grazing strategies

The development of IMF accumulation in beef cattle can be impacted by grazing techniques, feeding schedules, and feeding frequency. Changes in the nutritional value and quantity of fodder accessible to the cattle, grazing practices can affect IMF accumulation.^47^^,^^90–92^ Studies have indicated that, compared to continuous grazing, rotational grazing, in which cattle are frequently moved to new pastures, might raise IMF levels. Rotational grazing may enable cattle to graze on forage of higher quality, which contains more energy and nutrients that can be utilized for fat deposition. Timing and frequency of feeding can also affect the deposition of IMF in beef cattle.^47^^,^^90^^,^^91^ Feeding cattle in the late afternoon or evening as opposed to the morning can raise IMF levels. This could be because feeding in the afternoon or evening interferes with the animal’s normal circadian rhythm, which affects when hormones are released and when fat is deposited. IMF deposition may also be impacted by feeding frequency. According to studies, feeding cattle less frequently, for example, once a day as opposed to twice daily, can raise IMF levels. This may be because less frequent meals can result in higher insulin production, which can encourage the accumulation of fat.^48^

### Slaughter weight and age

Research has shown that the IMF content in cattle increases with age, and different slaughter ages affect the IMF accumulation in various breeds of cattle. Studies have found that as slaughter age increases, IMF accumulation also increases in breeds such as Wagyu, Angus, Hereford and Simmental.^93–95^ Additionally, the expression of various genes related to fat metabolism also changes with age. IMF content is also found to increase with slaughter weight in various breeds, and prolonged fattening periods can lead to higher IMF content but may also cause back fat to thicken, affecting yield grade. Therefore, only animals with a high genetic potential for marbling should undergo prolonged fattening.^96^^,^^97^

Furthermore, the relationship between IMF accumulation and slaughter weight is positive, and genetics, nutrition, and management can affect the variance in IMF composition between different slaughter ages and weights.^98–100^ The IMF content of various muscles as well as marbling score increased with increasing slaughter age in Angus, Hereford, and Wagyu x Angus crossbreds.^94^ However, prolonged fattening periods can lead to declining feed efficiency and thicker back fat, which can negatively impact yield grade.^98^ As age increases IMF accumulation also increases in most cattle breeds, but double-muscled Belgian Blue bulls do not display similar changes as they age.^101^

## Nutritional strategies

Improved IMF in beef cattle is mostly the result of nutrition.^102^ Feeding beef cattle a high-energy diet, employing feed additives, and adjusting feeding times, have been shown to improve IMF.^18^,^47^ By feeding finishing cattle a high-energy diet or by supplementing their diet with specific nutrients like vitamin E or conjugated linoleic acid, it is possible to improve the marbling of beef.^103^ Grazing, foraging, and nutrient supplements can be tailored to certain cattle breeds.

It is crucial to ensure that initiatives intended to increase output and expertise in the beef sector also have the strategic benefit of improved environmental outcomes, necessitating ongoing, thorough life cycle assessments.^91^ Nutritional practises have an impact on beef’s physical characteristics, nutritional makeup, and carcass composition. For example, the addition of betaine to the diet can change the distribution of fat in an animal’s body, which in turn affects carcass composition.^38^ Many dietary factors, such as fetal nutritional programming, concentrate to roughage ratios, grain processing, vitamin levels, feed additives, and stage-specific feeding schedules, might affect the deposition of IMF.^17^^,^^102^

### Dietary energy levels and fatty acid profile

The amount of dietary energy and fatty acid composition can both influence IMF deposition in beef cattle. Dietary energy content has a big effect on IMF deposits.^45^^,^^104^ In contrast to cattle fed low-energy diets, IMF levels are higher in cattle fed high-energy diets because they provide more nutrients and energy that can be used to retain fat.^38^^,^^105^ The fatty acid makeup of the feed may potentially have an impact on IMF deposition. Feeding cattle diets high in unsaturated fatty acids, like those found in flaxseed or soybean oil, may cause IMF levels to rise, in contrast to diets heavy in saturated fatty acids.^106^^,^^107^ The reason for this is enhanced IMF deposition is brought on by the easier incorporation of unsaturated fatty acids into adipose tissue. Dietary fibre type can affect IMF deposition in addition to the fatty acid profile. In contrast to diets high in indigestible fibre, such as maize stover, feeding cattle diets high in digestible fibre, such as beetroot pulp, can raise IMF levels.^108^ Producers can maximize IMF deposition by offering feeds that are high in energy, have the right fatty acid profile, and contain the right kind of fibre.

In general, according to a number of studies, beef calves on high-concentrate diets had more IMF than those fed low-concentrate diets. This is because feeding cattle concentrates like rice, barley, corn, and cassava at high levels raises net energy, which is necessary for fat growth. For instance, Angus crossbred steers finished on concentrate had more IMF than those finished on grass, and high-concentrate diets raised the IMF content of Hanwoo and Wagyu steers. Further, fattening diets with a high concentrate-to-roughage ratio influenced the expression of adipogenic genes in fat depots resulting in higher marbling scores due to accelerated differentiation of intramuscular preadipocytes.^108–110^

### Feed additives

Enhancing the IMF composition of beef cattle can be accomplished by using feed additives. In order to increase the IMF content of beef, metabolic modifiers can increase the growth rate, feed efficiency, and IMF composition of beef cattle. The use of permitted modifiers can help preserve acceptable IMF content.^111^^,^^112^ Feed additives have been found to have an impact on beef cattle marbling deposition. The use of feed additives, such as phytogenic feed additives, has been shown to improve carcass traits, including marbling score. In a study by Filho et al., bulls fed a blended phytogenic feed additive had greater hot carcass weight than the control group, with no differences in marbling score.^113^ Additionally, in another study by Castro Ferraz Júnior and Carvalho, feed additives were found to increase efficiency in energy and protein metabolism, which could potentially contribute to improved marbling deposition.^114^ These findings suggest that the use of feed additives, particularly phytogenic feed additives, may have a positive impact on beef cattle marbling deposition.

Furthermore, direct-fed microbial additives containing live beneficial microorganisms like yeast and strains of Lactobacillus, can improve intake and growth performance in calves.^115–117^ While beta-agonists are rarely used in broad pasture systems,^118^ there are other feed additives that can modify the IMF composition in beef cattle. For instance, conjugated linoleic acid (CLA), a fatty acid that occurs naturally and has been found to promote human health by reducing the chance of acquiring cancer and cardiovascular disease. Feeding CLA to beef calves can enhance the proportion of advantageous fatty acids, such as omega-3 fatty acids.^119^

Another feed ingredient that can impact IMF composition is vitamin E. This antioxidant has been shown to enhance the oxidative stability of beef, which can affect its sensory attributes and shelf life. Feeding beef calves vitamin E can enhance the amount of unsaturated fatty acids in IMF, improving the nutritional quality of the meat.^120^ Other feed additives that have been found to impact IMF composition include fish oil, linseed, and soybean oil.^35^ These additives can reduce the amount of saturated fatty acids in IMF while increasing the amount of good fatty acids like omega-3 fatty acids. By choosing the right feed additives, it is possible to significantly influence the composition of IMF in beef cattle, offering consumers a healthier and more nutritious product with enhanced flavour.

Both probiotics and prebiotics can enhance the IMF in beef cattle by fostering the development and activity of good gut bacteria and by improving nutrient utilization.^121–123^ Probiotics are living microbes that can colonize the gut and balance the microbiota. They can increase the fermentation of nutrients by stimulating the growth of advantageous gut bacteria result in improved IMF composition and feed efficiency.^124^^,^^125^ This can lead to higher feed efficiency and IMF composition in beef cattle. Prebiotics can improve nutrient utilization and IMF composition in beef cattle by establishing a healthy gut flora.^125^ Prebiotics can increase the growth and activity of good gut bacteria, resulting in better gut health and nutrient absorption. By encouraging a healthy gut microbiota and enhancing nutrient utilization, prebiotics and probiotics can be helpful tools in enhancing the IMF composition of beef cattle.^121–123^

### Hormone implants and regulations

In order to encourage growth and increase feed efficiency, hormone implants are frequently used in the production of beef cattle.^126^These implants contain synthetic testosterone and estrogen, which have been shown to increase IMF and marbling in beef cattle.^127^^,^^128^ To obtain IMF accumulation with hormone implants, however, it may be necessary to increase dietary calorie intake and fatty acid composition. Implants are categorized according to their potency, with higher-potency implants comprising trenbolone acetate and estrogenic hormones. While the use of implants can increase production and growth rate, farmers are worried about a potential decline in carcass quality grade, particularly in terms of marbling score, which is the most important component in determining quality grade.^126^^,^^129^

The quality of fresh meat is not adversely affected by hormone implants when they are handled properly.^130^ Growth-promoting implants can increase average daily gain, feed efficiency, carcass weight, and ribeye area while decreasing greenhouse gas production, water use, and land use.^131–134^ This has both economic and environmental advantages. However, the success of implants depends on a number of variables, including dietary needs, breed, developmental stage, and reaction to various implant types.^135^ Although beef cattle with implants may reduce less tender steaks than those without them, overall consumer satisfaction with meat from implanted calves remains high.^130^ In conclusion, the use of hormone implants in the production of beef cattle can have both favourable and unfavorable effects on the quality of the meat, but when used correctly, they can increase productivity and sustainability in the beef sector without significantly degrading the quality of the meat.^130^

The deposition and composition of IMF in beef cattle are significantly influenced by hormonal control. The uptake and storage of glucose and fatty acids in muscle tissue, which are promoted by insulin, are essential for IMF deposition. Other hormones, such as growth hormone, androgens, and glucocorticoids, also have an impact on this process.^38^^,^^136^ The effect of hormonal control on the accumulation of IMF in beef cattle is summarized in the Table 1. IMF formation in beef cattle is mostly regulated by hormones, with multiple hormones interacting to either encourage or hinder fat deposition. The composition of IMF in beef cattle can also be impacted by the use of hormones. For example, Estradiol-17 β (E2) is among the hormones that are most frequently used in the raising of beef. Beef cattle can produce more IMF when given E2 supplements.^137^^,^^138^ The composition of IMF in beef cattle can, in general, be significantly influenced by hormone supplementation. While the use of hormones can increase muscle mass and promote growth, it is important to consider the potential effects on the quality and healthfulness of the meat produced.

**Table 1.** The effect of hormonal control on the accumulation of IMF in beef cattle.

Hormone	Effect on IMF accumulation	References
Insulin	Increases IMF levels by promoting glucose and fatty acid uptake and storage in muscle tissue.	^139^
Growth hormone	Increases IMF levels by promoting the differentiation of preadipocytes into mature adipocytes	^140^
Androgens (e.g., testosterone)	Increases IMF levels by promoting muscle growth and development.	^136^
Glucocorticoids (e.g., cortisol)	Can increase or decrease IMF level depending on the level of exposure; high levels can lead to increased fat deposition in muscle tissue.	^38^

### Vitamin and antioxidant dietary supplements

The accumulation of IMF in beef cattle has been demonstrated to be positively impacted by antioxidant and vitamin supplementation. Antioxidants, such vitamin E and selenium, have been demonstrated to raise meat quality by lowering oxidative stress and enhancing meatcolor and flavor.^141^ Antioxidant supplements have also been demonstrated in studies to raise IMF levels in beef cattle. For instance, adding vitamin E to feedlot and grazing cattle increases IMF levels.^17^ Selenium administration raises IMF levels in grazing cattle.^142^ Vitamins like vitamin D and vitamin A affect the accumulation of IMF in beef cattle. As a result of its function in controlling the metabolism of calcium and phosphorus, vitamin D raises IMF levels in feedlot cattle.^143^ Due to its part in controlling lipid metabolism, vitamin A has also been found to raise IMF levels in beef cattle.^5^^,^^38^ In general, supplementing with vitamins and antioxidants can help prevent the deposition of IMF in beef cattle. By providing the right amounts of antioxidants and vitamins to their cattle, producers may maximize IMF deposition.

Dietary supplementation with resveratrol, a type of antioxidant, increased beef antioxidant capacity and improved meat quality under high-oxygen packaging conditions.^144^ Supplementation with a mix of vitamins and fat-soluble vitamins (ADE) improved antioxidant status and meat quality in Nellore cattle.^145^ Maternal vitamin and mineral supplementation during pregnancy increased trace mineral concentrations in the liver of beef heifers and their foetuses, potentially improving immune system function in offspring.^146^^,^^147^ Dietary supplementation with flavonoids improved animal performance, nutrient digestibility, antioxidant status, and the quality of meat and milk in cattle.^148^ Supplementation with selenium and vitamins A, D, and E improved the implantation ability and reproductive parameters in heifers undergoing embryo transfer.^149^ These findings suggest that vitamin and antioxidant dietary supplements can have beneficial effects on beef cattle, improving their antioxidant capacity, meat quality, reproductive performance, and overall health.

## Emerging technologies

### Genomic selection

Genomic selection represents an innovative strategy for enhancing intramuscular fat (IMF) composition in beef cattle. Through the utilization of advanced genomic tools, breeders can pinpoint specific genetic markers associated with favourable traits such as marbling, facilitating precise and well-informed decisions within breeding programs.^150–152^ This technique offers the potential for swift genetic advancements, optimizing the production of high-quality beef.^153^^,^^154^ It involves analysing extensive genomic data to calculate genomic estimated breeding values (GEBVs), which predict the genetic potential of each animal for traits such as marbling. This capability enables early and precise evaluations of genetic worth, thereby reducing the generation interval and accelerating the dissemination of desirable characteristics.^153^^,^^155^

Unlike traditional breeding, which relies on observable traits, genomic selection provides insights into the genetic factors influencing these traits. This proactive strategy helps breeders shape the genetic makeup of their herds more effectively.^156^ Studies have identified key genes and pathways regulating IMF deposition, such as COL4A1, TCAP, *MYH7*, and *PCK1.*^157^^,^^158^ Differential expression of genes involved in lipid metabolism has also been observed between different fat depots in cattle (Hudson et al. 2020). Genomic selection has shown promising results, improving prediction accuracy and genetic gain in beef cattle.^153^^,^^154^ It has been effective in identifying loci under selection for traits like fertility and carcass merit in various breeds.^159^^,^^160^

### Genome editing

Genome editing serves as a potent tool for modifying the genetic composition of animals, offering substantial potential for augmenting IMF accumulation in beef cattle.^161–164^ Advanced technologies such as CRISPR/Cas9, TALENs, and ZFNs facilitate precise DNA alterations, making them suitable for improving meat quality characteristics such as IMF levels.^165–169^ CRISPR/Cas9, in particular, is widely embraced due to its simplicity and effectiveness.^170^Current research endeavours are focused on refining these technologies, addressing specific benefits and challenges.^163^^,^^168^ Discussions within scientific and regulatory circles emphasize ethical considerations and responsible implementation.^166^ By precisely modifying DNA sequences, genome editing holds the potential to develop cattle with increased IMF content and reduced environmental impact, thereby enhancing productivity.^171^^,^^172^ For example, the inactivation of the myostatin gene in Belgian Blue cattle has been shown to boost muscle mass and improve IMF deposition.^173^^,^^174^

Genome editing significantly reduces the time required to select for target traits by quickly modifying important genes challenges such as maintaining genetic diversity and avoiding inbreeding.^175^^,^^176^ Studies have demonstrated the effectiveness of CRISPR/Cas9 in enhancing IMF composition, targeting genes involved in fat metabolism such as *FASN*, PPARγ, *FABP4* and *MSTN.*^177^ This technology can also help to enhance disease resistance, reproductive traits, and overall animal production to meet the growing demand for animal food and protein.^168^^,^^178–180^ In addition, CRISPR/Cas9 technology shows promise as a valuable tool for enhancing IMF deposition in beef cattle by precisely editing genes involved in fat metabolism and deposition. Studies have targeted genes such as *FASN, PPARγ, FABP4*, and *MSTN* to increase IMF content in cattle.^177^ This advanced genome-editing technology surpasses previous methods to help fulfill the increasing demand for animal food and protein.^168^^,^^178–180^

**Figure 1.**
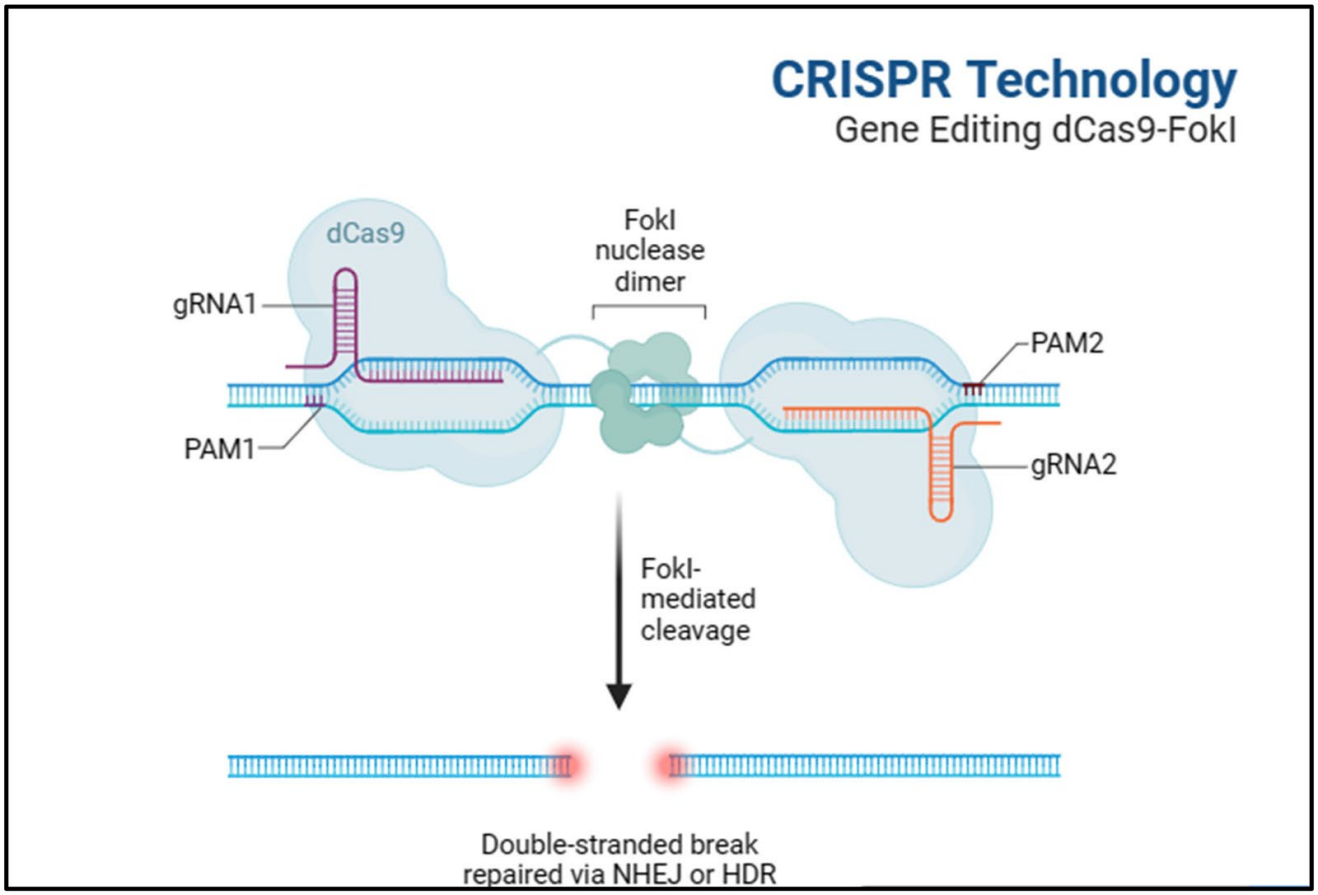
Application of CRISPR-Cas9 technology in gene editing.

Figure 1 presents a general overview of the CRISPR technology, specifically the "Gene Editing dCas9-FokI" system. It shows relevance for enhancing IMF deposition in beef cattle. It shows the key components involved in this technology, including the FokI nuclease dimer responsible for DNA cleavage and its interaction with the modified dCas9 protein. The figure also highlights the presence of two guide RNAs (gRNA1 and gRNA2) and their corresponding protospacer adjacent motifs (PAM1 and PAM2). These elements play a crucial role in guiding the dCas9-FokI complex to specific target genes associated with IMF deposition. Once the complex is bound to the target gene, the FokI enzyme is activated, leading to DNA cleavage at the site referred to as ‘FokI mediated cleavage.’ Moreover, the figure illustrates the repair process for the double-strand breaks induced by the DNA cleavage. It showcases two potential repair mechanisms: non-homologous end joining (NHEJ) and homology-directed repair (HDR). These repair pathways enable the cell to fix the DNA damage and introduce desired genetic modifications that can enhance IMF deposition. Generally, Figure 1 presents the CRISPR/Cas9 technology, specifically the ‘Gene Editing dCas9-FokI’ system, as a powerful tool for targeting and modifying genes associated with IMF deposition in beef cattle. This technology holds promise for improving the quality and quantity of IMF, thereby enhancing the desirable characteristics of beef cattle.

### Epigenomics

Epigenetic changes are molecular mechanisms with complicated features that require close analysis in the control of IMF deposition in beef cattle.^12^^,^^20^^,^^181^^,^^182^ These intricate molecular modifications entail heritable alterations in gene expression without altering the DNA sequence, a phenomenon extensively studied in various biological contexts.^183–185^ Comprehensive exploration of epigenomic dynamics necessitates discerning the interplay between genetic predispositions, environmental influences, and specific genomic loci undergoing epigenetic modifications.^186–188^ Advanced techniques such as high-throughput sequencing and chromatin immunoprecipitation are essential for mapping and analysing these epigenetic markers throughout the bovine genome, especially in areas related to IMF regulation ^182^^,^^189^^,^^190^

Epigenomic studies have provided a deep understanding of the molecular basis for intramuscular fat deposition in beef cattle. By using comparative epigenomics, genome-wide association studies, and selection signature analyses, researchers have revealed tissue-specific epigenetic patterns linked to economically important traits.^74^^,^^191^ DNA methylation patterns in critical tissues like the liver and longissimus dorsi muscle have been associated with IMF deposition, highlighting the significant role of epigenetic changes.^192^ Additionally, epigenetic modifications have been found to be involved in transcriptional regulation and function in complex traits variation and adaptive evolution, particularly in relation to reproduction traits.^39^ The integration of epigenomic information with traditional and molecular QTL analysis has also identified specific genes and genomic regions that are associated with reproduction traits and sperm motility.^193^ Generally, epigenomic studies have provided valuable insights into the regulatory mechanisms underlying IMF deposition and have the potential to inform genomic improvement programs in beef cattle.

### Microbiome manipulation

In the pursuit to improve IMF composition in beef cattle, attention has turned toward the manipulation of the microbiome, the diverse collection of microorganisms residing in the digestive systems of these animals.^194–200^ This intricate biological system is crucial for processing nutrients and facilitating their absorption. As a result, scientists are investigating ways to modify these microorganisms and their effects on the accumulation of fat in muscle tissue.^196–200^

The main goal of microbiome manipulation is to improve feed efficiency in cattle, prioritizing the allocation of more resources toward intramuscular fat deposition and enhancing the desired marbling in beef.^201–206^ This strategic method is in line with sustainability objectives and enhances the conversion of feed into high-quality meat products, presenting potential advantages for both the industry and the ecosystem.^201–206^

Microbiome manipulation is becoming an increasingly promising frontier in cattle agriculture, incorporating comprehensive approaches that encompass animal nutrition, health, and the microbiome to effectively improve beef quality in a sustainable manner.^207^ This paradigm shift in livestock management practices warrants further exploration and application, shaping the trajectory of beef production.^208^^,^^209^

Additionally, manipulation of the microbiome has been shown to effectively increase marbling content in cattle in various circumstances. Dietary approaches, such as high-energy grain diets, have proven more effective than pasture diets in achieving higher marbling.^17^ Within grain-based feedlot diets, maize outperforms barley, and barley diets are superior to sorghum ones.^210^ Moreover, steam flaking improves marbling, particularly with sorghum compared to dry-rolled grain.^211^ Genetic selection methods have also been investigated and revealed associations between intramuscular fat and characteristics like marbling, growth and tenderness. This led to the identification of advantageous alleles for marbling as direct genetic markers.^98^ These findings highlight the potential of manipulating the microbiome to enhance marbling content in cattle.

### Transcriptomic profiling

Transcriptome analysis, enabled by advanced sequencing methods, has transformed our comprehension of the molecular mechanisms responsible for intramuscular fat composition in beef cattle.^212^^,^^213^ This methodology helps explain the complex biological processes involved in fat metabolism and accumulation by concurrently examining the expression of numerous genes.^158^ By not only capturing individual gene expression levels but also elucidating the interactions and regulatory networks among genes, transcriptomic profiling offers valuable insights into the coordinated molecular processes propelling IMF accumulation.^214^^,^^215^

One of the primary benefits of transcriptomic profiling lies in its capacity to reveal new genes and pathways that impact intramuscular fat composition in beef cattle.^181^ Through the comparison of transcriptomes from animals with varying fat qualities, scientists can detect genes showing differential expression linked to superior fat traits.^216^ These genes may potentially encode controllers of adipocyte differentiation, signalling molecules, or enzymes involved in lipid metabolism. This discovery presents hopeful avenues for future investigation and potential modification through genetic selection. Transcriptomic analysis can elucidate alternative splicing processes and post-transcriptional regulations affecting fat-related variables, thereby enhancing our understanding of fatness traits.^212^ Through the discovery of distinct splicing patterns linked to fat characteristics, transcriptomic data analysis reveals isoforms that may play specific roles in adipogenesis or intramuscular fat metabolism.^216^

Integrating transcriptomic profiling with other genomic data, like genetic markers or epigenetic details, offers a more comprehensive insight into the genetic framework of obesity traits.^212^ When transcriptomic data is merged with genetic marker information, it becomes possible to pinpoint candidate genes that show differential expression and are genetically linked to fat-related characteristics. This approach provides valuable clues about the underlying genetic determinants of fat traits and may uncover potential genetic markers for selecting animals with superior fat qualities.^21^

Transcriptomic profiling studies in beef cattle have provided valuable insights into the factors controlling IMF deposition. Several studies have identified key genes and regulatory polymorphisms associated with IMF content. For instance, two gene modules significantly associated with fat deposition and identified three hub genes (*TCAP*, *MYH7*, and *TNNC1*) that may regulate bovine IMF deposition.^74^ A genome-wide association analysis and identified multiple expression quantitative trait loci (eQTL) regulating the expression levels of genes associated with IMF.^217^ Priyanka Banerjee et al. used RNA-Seq data to predict the future reproductive potential of beef heifers and identified differentially expressed genes associated with reproductive outcomes.^218^ Gustavo Schettini et al. identified potential regulatory genes associated with the essential fatty acid profile in beef cattle, including *ECHS1, IVD, ASB5,* and *ERLIN1.*^219^ Tianliu Zhang et al. characterized region-specific expression differences in beef cuts and identified candidate genes related to meat quality, including *TNNI1, TNNT1, SCD, LPL, ALDH2, IVD, ACADS, PHPT1, SNTA1, SUMO1, CNBP, CDC37, GAPDH, NRBP1, ATP8, COX8B*, and *NDUFB6.*^220^

### Metabolomics

The complicated metabolic mechanisms offer the composition of the IMF in beef cattle are better understood with the use of metabolic profiling.^181^ Metabolomic profiling offers a thorough perspective of the metabolic pathways and biomarkers connected to fat metabolism and deposition by examining and measuring the small-molecule metabolites present in biological samples ^39^^,^^181^

One of the key advantages of metabolomic profiling is its ability to capture dynamic changes in metabolite concentrations and fluxes in response to various physiological and environmental circumstances.^221^ Researchers can distinguish metabolites associated with superior fat qualities by comparing the metabolomes of animals with different types of fat. These may include acylcarnitines, phospholipids, and fatty acids, shedding light on the metabolic processes that drive fat accumulation.^221^^,^^222^

Moreover, metabolomic profiling can find novel biomarkers that represent the composition of IMF, which can be utilized for effective selection and breeding programs.^45^^,^^223^ By incorporating metabolomic data are merged with other omics information, such as genomic and transcriptomic data, scientists can uncover the genetic and molecular basis of fat properties.^181^^,^^224^ Sophisticated statistical and bioinformatics techniques are utilized to examine metabolomic data, facilitating the detection of patterns in metabolites linked to fat traits and provides the enhancement of metabolic pathways through variations in metabolite abundance.^225^^,^^226^

Metabolomic profiling provides valuable insights into the biomarkers and metabolic pathways associated with IMF in beef cattle, improves our understanding of fat metabolism and providing valuable resources for management, breeding, and selection strategies to enhance the IMF composition of beef cattle.^227^

Numerous research efforts have utilized metabolomic analysis to examine the influences of various metabolites on intramuscular fat in beef cattle. For example, the early castration of Holstein cattle was observed to alter hepatic metabolites and associated pathways, leading to advancements in beef marbling production and heightened functional quality.^228^ Another study on Charolais heifers found that changes in branched-chain amino acid metabolism and body composition were associated with differences in feed efficiency measured as a residual feed intake (RFI).^229^ Similarly, a comparison of white and yellow fat depots in crossbred cattle and identified key metabolites associated with beef fat colour, providing potential biomarkers for the selection of fat colour in live animals.^230^ Metabolomic analysis has also been applied to measure dynamic metabolic responses and understand the genetic architecture of various cattle traits, providing valuable information for further research and applied breeding programs.^231^ Generally, these studies highlight the importance of metabolomic analysis in understanding the metabolic mechanisms underlying IMF and other economic traits in beef cattle.

**Figure 2.**
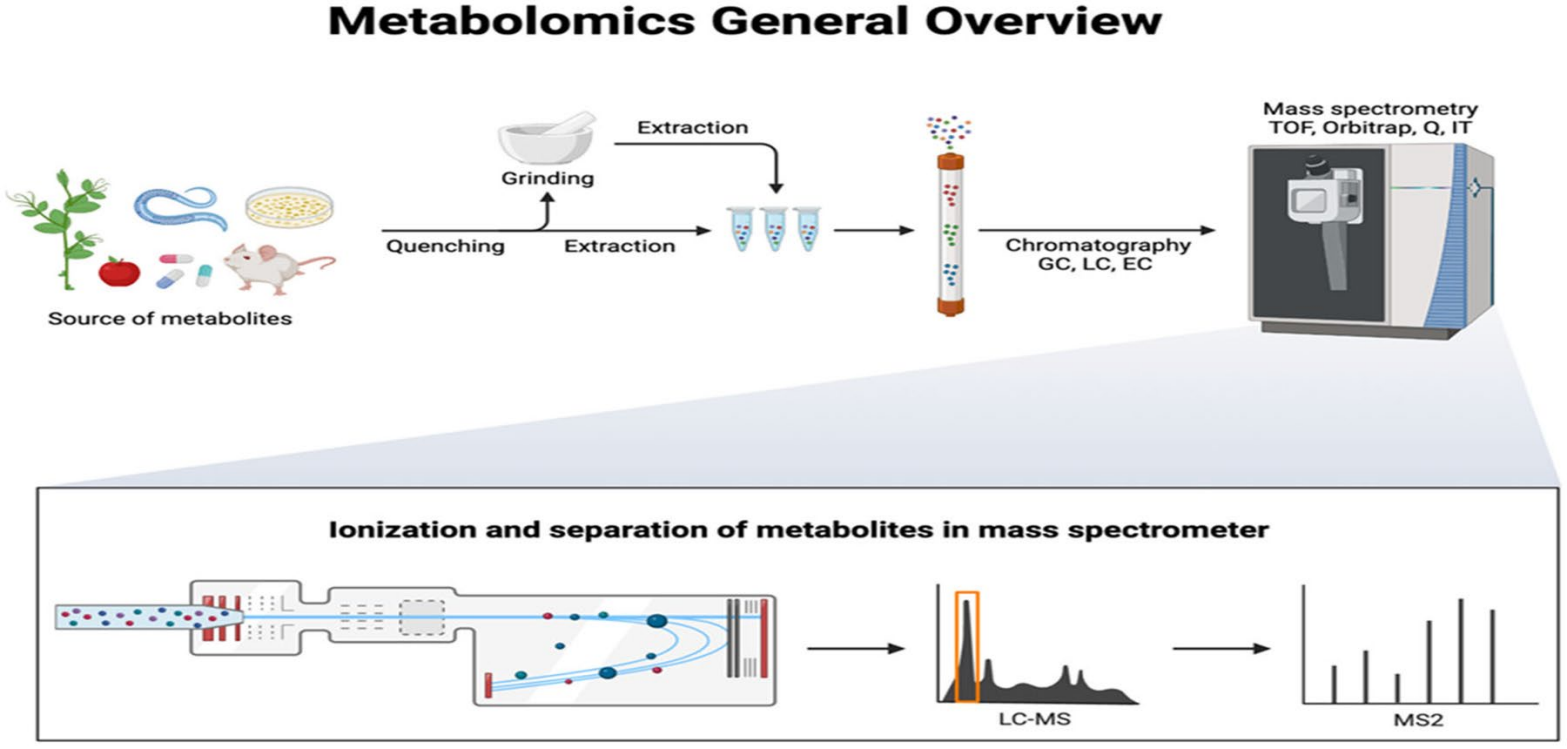
General overview of metabolomics analyses.

Figure 2 offers a concise overview of how mass spectrometry can be employed for metabolomic analysis. The process entails preparing the sample, ionizing the molecules within it, separating the resulting ions based on their mass-to-charge ratio, and subsequently detecting them. The data obtained from this analysis enables the determination of both the identity and quantity of metabolites present in the sample.

## Challenges and limitations of strategies to improve the IMF

For beef cattle, increasing IMF is crucial for improving meat quality and consumer acceptance. Strategies intended to improve IMF in beef cattle, however, are accompanied by a number of difficulties and restrictions. Some breeds of beef cattle have little genetic potential for IMF, and it can be difficult and time-consuming to improve IMF through selective breeding. In other breeds, such as Wagyu animals chosen for higher degrees of marbling have demonstrated increased IMF levels, indicating the effectiveness of genetic selection for enhanced IMF.^38^^,^^232^^,^^233^ IMF levels may also be impacted by the quantity and nature of feed given to cattle. But excessive fat deposition brought on by overfeeding can reduce the quality of meat. The type of feed used can influence the fatty acid makeup of meat, which can affect customer acceptance.

Furthermore, environmental factors such as temperature, humidity, and stress can have an effect on IMF levels.^17^ The levels of IMF in beef cattle, for instance, have been demonstrated to decrease during heat stress ^86^ and they can increase with implantation of growth-promoting hormones. These procedures, however, are debatable, and some customers might not find them acceptable.^234^ Some methods of boosting IMF, such as genetic selection and feed supplementation can be expensive and may not be economically viable for many farmers. In conclusion, raising IMF levels in beef cattle is a challenging process with a number of constraints. To increase IMF levels and enhance meat quality, a combination of genetic selection, dietary management, and environmental control may be useful.^18^

## Implications

Enhancing intramuscular fat composition in beef cattle through emerging technologies, nutritional strategies, and improved management practices holds significant implications across the industry. This approach not only elevates the overall quality of beef by enhancing tenderness and flavour but also presents an economic advantage for producers because higher-quality products often command a premium in the market. Moreover, meeting consumer preferences for well-marbled beef allows producers to differentiate their products, potentially gaining a competitive edge and fostering consumer loyalty.

On a broader scale, the adoption of these strategies contributes to the modernization of the beef industry by embracing technological advances and promoting more research. The focus on sustainable management practices underscores a commitment to animal health and welfare, aligning with ethical considerations. Additionally, the environmental impact is addressed through potential improvements in production efficiency and resource optimization, supporting responsible practices in cattle farming. Overall, this comprehensive approach not only benefits producers and consumers but also promotes a more sustainable and competitive beef industry.

## Future perspectives and directions

Finding certain genes and genetic markers linked to IMF deposition and composition will require more study. The creation of breeding programs that prioritize selecting animals with desirable IMF features may result from this. IMF deposition may be enhanced by improved management techniques that lessen stress and enhance animal well-being. Changes to handling and living arrangements may be part of this. Additional research is required to understand the function of hormones in IMF metabolism and to create methods for controlling hormone levels in cattle to enhance IMF composition. Understanding the effects of environmental conditions such as temperature and light on IMF deposition and composition is necessary to meet consumer demands for preferences for beef with particular amounts of IMF. To improve IMF composition and satisfy consumers’ shifting wants and tastes, there is a need for continuous research and innovation in the sector of beef cattle production.

## Conclusions

The main aim of this paper was to provide insights into emerging technologies, nutritional practices, and management strategies to improve IMF composition in beef cattle. This review highlighted the importance of genetics, nutrition, and management strategies to enhance IMF accumulation in beef cattle. The significance of IMF deposition to enhance meat taste and tenderness underscores its importance for consumer desirability. Genomic selection and editing, coupled with proper management and nutritional approaches offer promising avenues to boost IMF deposition across various cattle breeds. For producers, adopting these strategies has the potential to enhance beef quality and overall profitability. To navigate the dynamic landscape of beef production, it is crucial for producers to recognize the influence of these factors on IMF composition and implement tailored strategies accordingly. Furthermore, a holistic perspective that incorporates emerging technologies, nutritional information, and effective management practices while considering environmental sustainability is paramount for the beef industry. While this review provides valuable insights into the current state of research on IMF accumulation in beef cattle, there is still much to be explored. Future research should focus on identifying gaps in current knowledge and emerging technologies that hold promise for optimizing IMF deposition. Achieving the ideal IMF composition in beef cattle necessitates a comprehensive and well-informed approach. As the industry evolves, ongoing research and collaboration will be crucial to determining the optimal synergy of genetics, nutrition, and management practices for maximizing IMF accumulation and ensuring the continued success and sustainability of beef production. By embracing emerging technologies and staying informed about nutritional and management advancements, producers can position themselves for success in the dynamic and competitive beef industry.

## Data Availability

All data used to write this article is available with corresponding author upon reasonable request
